# Lung isolation using retrograde intrabronchial Fogarty catheter: Experience from 35 cases

**DOI:** 10.1016/j.xjtc.2025.09.006

**Published:** 2025-09-17

**Authors:** Najim Hanna Hirmiz, Abdulsalam Yaseen Taha

**Affiliations:** a Department of Cardiothoracic and Vascular Surgery, Basra Teaching Hospital, Basra, Iraq; b Department of Cardiothoracic and Vascular Surgery, College of Medicine, Al-Nahrain University, Baghdad, Iraq; c Department of Thoracic and Cardiovascular Surgery, College of Medicine, University of Sulaimani, Sulaimani, Region of Kurdistan, Iraq

**Keywords:** one-lung ventilation, Fogarty catheter, retrograde technique

## Abstract

**Objectives:**

One-lung ventilation is vital in thoracic surgery to improve exposure and protect the contralateral lung. Standard techniques using double-lumen tubes or bronchial blockers require specialized equipment and training, which are often unavailable in emergencies, pediatric cases, or low-resource settings. Retrograde intraoperative insertion of a Fogarty catheter into an open bronchus is a seldom-reported alternative, with only 1 previous case documented.

**Methods:**

We present a case series of 35 patients (25 male, 10 female; age 4-50 years, mean 28 years) who underwent thoracic surgery in Basra and Baghdad hospitals between 1984 and 2000. Lung isolation was achieved by inserting a Fogarty catheter directly into the bronchus via the surgical field. Indications included pulmonary hydatid cysts (n = 19), thoracic trauma (n = 14), and bronchopleural fistulae (n = 2). The technique was first used during the Iraqi-Iranian war in 1984 on a wounded soldier requiring lobectomy and was most recently used in 2000 on a pediatric patient with a ruptured hydatid cyst.

**Results:**

One-lung ventilation was successfully achieved in all cases without technique-related complications. Oxygenation and ventilation remained stable throughout procedures, enabling safe and effective surgical management across various thoracic conditions. No adverse events from catheter placement were reported.

**Conclusions:**

This is the first case series—and only the second report—on retrograde Fogarty catheter insertion for lung isolation. The method is simple, safe, cost-effective, and especially useful in emergencies, pediatric surgery, and settings lacking standard equipment or trained personnel.

**Figure:**
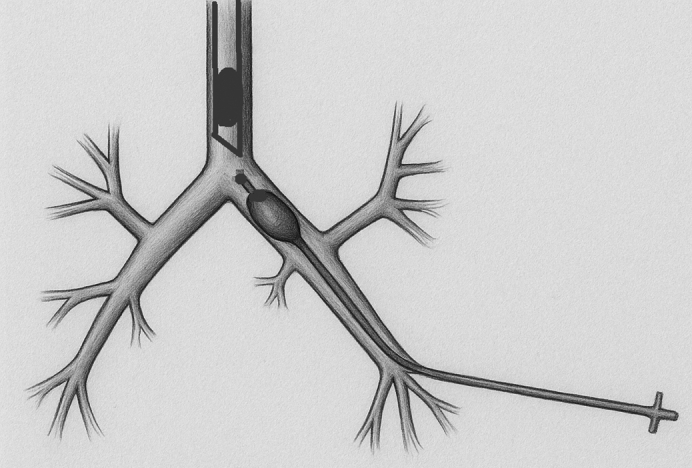
An illustration of the technique of intrabronchial Fogarty catheter for lung isolation.

**Central Message**

Retrograde Fogarty catheter insertion is a safe, simple, and effective alternative for lung isolation in emergencies, pediatric patients, and low-resource thoracic surgical settings.

**Perspective**

This is the first case series describing retrograde intrabronchial Fogarty catheter insertion for one-lung ventilation. It offers a practical, low-cost alternative when conventional tools are unavailable—especially in emergencies, pediatric patients, and austere environments—highlighting its potential global utility in thoracic surgery.

One-lung ventilation (OLV) is a critical component in thoracic surgical procedures, enabling optimal exposure of the operative field and protection of the contralateral lung. Standard techniques to achieve lung isolation typically involve the use of double-lumen endotracheal tubes (DLTs) or bronchial blockers, which require specific equipment, patient suitability, and anesthetist expertise in advanced airway management.^1^ However, these conventional methods may not always be feasible, especially in pediatric patients, in emergency settings, or in resource-limited environments where equipment and trained personnel may be unavailable.^2^

In such cases, alternative approaches to lung isolation are essential. One such method is the retrograde insertion of a Fogarty embolectomy catheter into the open bronchus to occlude the targeted lung. Originally introduced in 1962 by Dr Thomas J. Fogarty for the purpose of embolectomy and thrombectomy, the Fogarty catheter revolutionized vascular surgery by offering a minimally invasive method for removing blood clots.^3^ Its success in vascular applications led to its adaptation in other fields, and more recently, it has been explored for bronchial occlusion during thoracic surgery.^4^

Unlike the retrograde method, the antegrade insertion of Fogarty catheters—whether through the endotracheal tube or externally—is more commonly reported in the literature for achieving lung isolation.^5^, ^6^, ^7^, ^8^, ^9^ Nevertheless, the retrograde approach, although less frequently discussed, may serve as a valuable alternative, especially in emergency surgeries or when conventional airway techniques are not practical.

Although retrograde insertion of a Fogarty catheter for lung isolation has been previously described in a single case report,^10^^,^^11^ there has been no comprehensive evaluation or published case series examining its broader application—particularly in thoracic emergencies and among pediatric patients. Given the scarcity of literature on this subject, this study aims to explore the feasibility and clinical utility of the retrograde Fogarty catheter technique for lung isolation in a variety of thoracic surgical settings encountered at hospitals in Basra and Baghdad.

## Patients and Methods

This prospective study included 35 patients, aged 4 to 50 years, who underwent thoracic surgery using a novel bronchial blocking technique between 1984 and 2000. The cohort consisted of 25 male and 10 female patients, with a mean age of 28 years. Formal institutional review board approval and individual consent was not required for this study, because it is a retrospective case series involving patients who were managed more than 4 decades ago.

The first case in this series involving intraoperative retrograde insertion of a Fogarty catheter for lung isolation was performed on April 8, 1984, at Basra Teaching Hospital. The patient was a wounded soldier from the Iraqi-Iranian War who required a right upper lobectomy because of a severe upper lobe injury. The most recent case took place on August 30, 2000, in a private hospital in Baghdad and involved a child with a ruptured hydatid cyst requiring a right middle lobectomy. Thus, the series spanned 16 years and was conducted across hospitals in both Basra and Baghdad.

The technique was initially adopted during wartime in Basra, as described by the operating surgeon (the first author of this paper). During this period, numerous anesthetists from across the country were mobilized to manage mass casualties. However, many had limited experience with the placement of DLTs, which, if incorrectly inserted, could result in serious complications during thoracic procedures. In response, the retrograde Fogarty catheter method was introduced as a safer alternative. Although it was first used in trauma cases, its application later expanded to other indications, as described in this article.

After the induction of general anesthesia, all patients were intubated with a single-lumen endotracheal tube and underwent thoracotomy. A Fogarty catheter was inserted under direct vision into the target bronchus during a brief period of apnea, after which ventilation was resumed. In children, catheters sized 3 to 5 Fr were used, while in adults, 6- to 9-Fr catheters were selected. In trauma and bronchopleural fistula cases, the bronchus was already open, whereas in pulmonary hydatid cysts, the cyst was excised first and an exposed bronchus within the cavity was chosen for catheter placement. The balloon was then inflated to achieve selective blockade of either the entire lung or an individual lobe, depending on the surgical requirement, and lung isolation was confirmed by the absence of ventilation in the target lung or lobe. After catheter removal, the bronchotomies were closed using interrupted polypropylene sutures.

This method was primarily used under specific conditions: emergency surgeries, unavailability or malfunction of double-lumen tubes, pediatric cases in whom DLTs were unsuitable, mispositioned or ineffective DLTs, surgeon preference due to limited familiarity with DLT systems, or the need for selective lobar isolation. Although not intended to replace conventional double-lumen tubes, the Fogarty catheter technique served as a practical and effective alternative when standard methods were unavailable or inappropriate.

## Results

Patients were categorized according to the underlying surgical indication as shown in Table 1. The Fogarty catheter bronchial blocking technique proved to be a reliable and effective alternative to traditional lung isolation methods. It was successfully used in all 35 patients without major complications. The procedure allowed for clear operative fields and adequate isolation, even in complex cases such as hydatid cysts and bronchopleural fistula.

**Table 1:** Surgical indications

Indication	Male patient	Female patient	Total
Hydatid cysts (children)	8	5	13
Hydatid cysts (adults)	3	3	6
Thoracic trauma	13	1	14
Bronchopleural fistula	1	1	2
Total	25	10	35

Key advantages included ease of use in both elective and emergency settings, suitability for pediatric patients in whom DLTs are not feasible, the ability to provide selective lobar isolation when necessary, and serving as an effective alternative in cases in which DLTs are unavailable, improperly positioned, or when the anesthetist lacks experience with their use.

## Discussion

OLV is a critical anesthetic and surgical technique that facilitates a collapsed, motionless lung on the operative side while maintaining adequate ventilation and oxygenation on the contralateral side. The primary purpose of OLV is to provide optimal surgical exposure and reduce the risk of contamination or injury to the nonoperative lung during intrathoracic procedures. It is widely indicated in thoracic surgeries such as pulmonary resections, esophagectomies, thoracic aortic repairs, and procedures involving the mediastinum or spine. OLV is also essential in managing conditions like bronchopleural fistulas, unilateral lung infections, and massive pulmonary hemorrhage, where isolation of 1 lung is necessary for patient safety and procedural success.^2^

The evolution of OLV techniques reflects significant advances in anesthetic practice and thoracic surgery over the past century. Initially, lung isolation was attempted using simple techniques such as bronchial occlusion with gauze or sponges. The introduction of the DLTs in the mid-20th century marked a major breakthrough, allowing selective ventilation and improved airway control. Since then, refinements in DLT design and materials, along with the development of bronchial blockers and video-assisted bronchoscopic guidance, have greatly enhanced the precision and safety of OLV. These advances have enabled anesthesiologists to manage complex thoracic cases more efficiently while reducing perioperative complications and improving patient outcomes.^1^

The retrograde intraoperative use of a Fogarty catheter for OLV through an open bronchus represents a novel yet underused approach to lung isolation in thoracic surgery.^10^^,^^11^ Traditional methods for OLV—such as DLTs, bronchial blockers, and selective endobronchial intubation—are the standard of care^12^^,^^13^ but may be infeasible in emergencies, pediatric populations, or in settings in which advanced airway tools and expertise are unavailable. In such scenarios, Fogarty embolectomy catheters offer a cost-effective and adaptable alternative, especially because of their availability in a wide range of sizes.^4^

This study highlights a novel and practical technique for achieving lung isolation intraoperatively in resource-limited settings. By using retrograde insertion of a Fogarty catheter through the surgical field, we demonstrated an effective alternative to conventional airway isolation methods. This approach proved especially useful in scenarios in which standard equipment or specialized anesthetic support was unavailable. Its simplicity, safety, and adaptability make it a valuable addition to the surgical toolkit for managing challenging thoracic cases such as pulmonary hydatid disease, trauma, and bronchopleural fistulae. To our knowledge, this is the first documented series using this method, underscoring its potential as a reliable option for selective lung ventilation in similar clinical environments.

A landmark case report by Ramesh and colleagues^11^ from the Freeman Hospital in the United Kingdom supports our findings and adds weight to the clinical utility of this method. In their patient with fibrotic lung disease undergoing off-pump left lung transplantation, single-lung ventilation failed abruptly after bronchial division, resulting in life-threatening desaturation. When conventional rescue strategies failed, the team innovatively inserted a size 10 Fogarty catheter retrogradely through the open left bronchus, achieving rapid reisolation of the lung. This maneuver avoided the need for cardiopulmonary bypass—which carries its own risks—and allowed the surgery to proceed. The catheter was subsequently removed through the bronchial suture line without complications. The authors note this method has been used successfully in multiple similar cases and underscore the value of preoperative computed tomography to size DLTs appropriately while also cautioning that this approach is anatomically unsuitable for right lung transplants.^10^^,^^11^

Our larger series complements the report from Ramesh and colleagues by demonstrating that this technique is not only effective in single complex cases but also scalable and feasible across a spectrum of thoracic conditions. Moreover, although Ramesh and colleagues used the technique as a rescue measure during transplantation, we applied it electively in several pediatric and emergency scenarios, confirming its broader versatility.

Additional reports in pediatric populations describe antegrade use of Fogarty catheters under bronchoscopic guidance, particularly in children younger than 8 years old, in whom conventional devices do not fit the airway.^7^^,^^8^ Although desaturation and bradycardia during placement have been reported in some studies,^8^ outcomes have generally been favorable. Our retrograde method avoids the need for bronchoscopic tools entirely and provides a direct, surgeon-controlled solution, which can be vital in low-resource or emergency settings.

It is also worth noting that the first reported case of retrograde intraoperative Fogarty catheter insertion for lung isolation in the international literature did not appear until 2014—several years after all the cases in our series had been completed by 2000. This highlights the originality of our early clinical experience and underscores the importance of documenting context-driven innovations, especially those developed in resource-limited or wartime settings.

Despite the technique's promise, it remains relatively rare and is best reserved for situations in which standard lung isolation strategies are unsuitable. It demands clear intraoperative coordination between the surgical and anesthesia teams and should be approached with careful case selection. Nonetheless, the absence of complications in both our series and the Freeman Hospital report suggests that when used judiciously, this method is safe, reproducible, and effective.^9^

Although our series was performed exclusively through open thoracotomy, we acknowledge that most pulmonary resections today are carried out by minimally invasive approaches. For these cases, standard DLTs or bronchial blockers remain preferable. Nevertheless, Fogarty catheters can be adapted for video-assisted thoracoscopic surgery or minimally invasive procedures, particularly in pediatric cases, by introducing Fogarty catheters through or parallel to a single-lumen endotracheal tube, with balloon position confirmed by fiberoptic bronchoscopy.^6^^,^^8^

In conclusion, the retrograde intraoperative use of a Fogarty catheter through the surgical field represents a valuable alternative for achieving OLV. Our case series demonstrates its success across a wide age range and a variety of indications, whereas the Freeman Hospital report illustrates its life-saving potential during unexpected failures of SLV in high-stakes procedures. Together, these findings highlight the Fogarty catheter's expanding role in thoracic surgery and call for further research to define its place within the armamentarium of lung-isolation techniques.

## Conflict of Interest Statement

The authors reported no conflicts of interest.

The *Journal* policy requires editors and reviewers to disclose conflicts of interest and to decline handling or reviewing manuscripts for which they may have a conflict of interest. The editors and reviewers of this article have no conflicts of interest.
